# Living with the incoherent: Practical insights on implementing European restoration policies for biodiversity policy integration

**DOI:** 10.1007/s13280-025-02180-2

**Published:** 2025-04-19

**Authors:** Fabian Pröbstl, Yves Zinngrebe, Michael Böcher, Sophia Schmid, Mathias Scholz, Barbara Stammel, Frank Hüesker

**Affiliations:** 1 Department Conservation Biology and Social-Ecological Systems, Helmholtz-Centre for Environmental Research (UFZ), Permoserstr. 15, 04318 Leipzig, Germany; 2 Department Systemic Environmental Biotechnology, Helmholtz-Centre for Environmental Research (UFZ), Permoserstr. 15, 04318 Leipzig, Germany; 3 Department of Political Science, Faculty of Humanities, Institute of Social Science, Otto von Guericke University Magdeburg, Zschokkestr. 32, 39104 Magdeburg, Germany; 4 Floodplain Institute Neuburg-Ingolstadt, Catholic University Eichstätt-Ingolstadt, Schloss Grünau, 86633 Neuburg an der Donau, Germany; 5 University of Applied Science Erfurt, Leipziger Straße 77, 99085 Erfurt, Germany

**Keywords:** Natura 2000, Policy coherence, Policy coordination, Public policy, River and floodplain restoration, Water framework directive

## Abstract

Policy incoherence is an omnipresent challenge in public administration. Focusing on the joint implementation of the European Water Framework Directive and the Habitats Directive, we analyze how administrative fragmentation hampers river restoration projects at the sub-national level in Germany. By shifting the analytical focus to institutional and administrative factors, we reveal barriers at institutional, processual and individual levels. We find that the failure of river restoration projects stems not only from practical hurdles, such as lack of resources, information, or manpower, but also from more fundamental flaws in the administrative arrangements. Based on a multi-stakeholder consultation, we argue that both bottom-up and top-down approaches can support better coordination and a local mandate for joint implementation. In particular, state-level planning can help reduce local coordination costs in the short term. The analysis highlights the critical role of individual actors and leadership, reframing and cross-sectoral coordination for policy integration.

**Supplementary Information:**

The online version contains supplementary material available at 10.1007/s13280-025-02180-2.

## Introduction

Europe records a significant loss of more than half of its pristine water-dependent ecosystems, such as rivers, streams, and floodplains (Ignar and Gygoruk 2015). These ecosystems provide vital ecosystem services, such as storing CO_2_ and providing habitat for endangered species (EEA 2020; Zingraff-Hamed et al. 2021). In response, the European Parliament enacted the European Nature Restoration Law in 2024, aiming to restore 20% of land and sea areas and 25 000 km of free-flowing rivers by 2030 (EU 2024). The European Water Framework Directive (WFD, 2000/60/EC) and the Habitats Directive (HD, 92/43/EEC) are two key pieces of underlying European legislation in this regard (Weigelhofer et al. 2020). In principle, both directives aim to improve the status of natural habitats and species conditions. However, the WFD also promotes more dynamic river systems, which may conflict with the HD if certain habitats or species are negatively affected. In a survey of 130 European experts, the HD was identified as the directive that most conflicted with the WFD in practice (Zingraff-Hamed et al. 2020). Effective river restoration requires coherence between these directives, but practical implementation faces challenges on the ground.

Water management is inherently cross-sectoral and much attention has been paid to the successful involvement of land managers, land availability and financial resources (Schröder et al. 2020). Studies have examined ecological, legal, and practical challenges but often overlook implementation structures at lower administrative levels (Boeuf and Fritsch 2016). In particular, lower water management and nature conservation authorities are crucial for the joint implementation (Schröder et al. 2020). Yet, they partly face different challenges than other stakeholders, such as fragmentation, policy incoherence, or poor coordination (Cejudo and Trein 2023a). Schröder et al. (2020) have started to explain weak coordination in terms of low physical proximity and high independence of the organizational units. However, the institutional challenges require further systematic analysis (Copetti and Erba 2024).

The debate on policy integration has identified administrative fragmentation as a source of ineffective implementation (Cejudo and Trein 2023a, b). Policy integration involves the alignment of sectoral policy goals, instruments, and implementation structures and can be defined as a process to develop coherent policies by promoting coordination across sectors and levels (Meijers and Stead 2004; Bornemann 2014; Persson and Runhaar 2018). Despite the identification of process-related enablers in terms of inclusion, integration, adaptation, and accountability (Zinngrebe 2018; Pröbstl et al. 2023; Runhaar et al. 2024), there are pressing gaps in explaining the lack of integration in terms of underlying structural causes (Persson and Runhaar 2018). A better understanding of these explanatory factors can be found in the public administration literature (Peters 2015; Bach et al. 2019; Molenveld et al. 2021), especially in the context of policy coordination.

In our study, we combine these perspectives to link the practical challenges of policy integration with an understanding of administrative dynamics and policy coherence. Thus, we analyze how institutional arrangements in Germany determine the ability of sub-national administrative authorities to jointly implement the WFD and HD as cross-cutting restoration policies. Germany is an interesting case due to its federal structures with advanced institutional and financial capacities. Yet, the country risks missing the proposed WFD targets for good ecological and chemical status by 2027 due to lack of land availability, public resources, and political support (Moss et al. 2020). Building on prior studies (Galler 2015; Schröder et al. 2020; Zingraff-Hamed et al. 2020), this article will broaden the scope by examining the joint implementation of the two directives based on the following questions:

1. How do the policy incoherencies between the European Water Framework Directive and the Habitats Directive manifest themselves at the sub-national administrative level in Germany?
2. Which factors support the positive coordination between water management authorities and nature conservation authorities for a coherent implementation of the two Directives?

The following sections provide theoretical insights into the administrative coordination, traditions, and joint implementation of the two directives in Germany. The methods section outlines our three-stage data collection approach, followed by the findings, which present the identified policy incoherencies from institutional, processual, and individual perspectives. We then discuss the underlying causes from a policy integration viewpoint, relate them to insights from public administration, and conclude with implications for multi-level governance.

## The German case study: Administrative coordination, traditions, and joint implementation of environmental directives

### Fragmentation and coordination in public administration

The growing complexity of modern socio-ecological problems necessitates greater specialization of individual administrative units (Jordan and Lenschow 2010; Lidén and Nyhlén 2023). This specialization, reinforced by the New Public Management, has resulted in fragmentation and selective focus of individual units, hindering the ability to address interdependent problems (Bogumil and Jann 2005; Molenveld et al. 2021).

Investing in decentralized coordination can mitigate this "siloism" by aligning decisions, reducing administrative overlap, and enhancing vertical communication. However, coordination is often expensive, complex, and time-consuming (Molenveld et al. 2021). As a result, many administrations practice negative coordination, where decision-making primarily considers others to avoid conflicts rather than to foster real collaboration. By contrast, actively seeking ways to work on solutions together (positive coordination), i.e., going beyond mere conflict avoidance, is the exception rather than the rule. This lack of coordination is not simply due to laziness, disinterest, or incompetence on the part of staff, as obstacles can be found at the institutional, processual or individual level (Bogumil and Jann 2005).

For example, formal and informal rules of administrative units prioritize internal actions, whereas external coordination is associated with higher security efforts and, thus, higher administrative costs (Peters 2015). These costs arise from different sources such as beliefs, power dynamics, turf battles, strategic performance metrics, or political influence (Peters 2018). Moreover, coordination demands during policy implementation are typically greater than during policy formulation. The prior requires real changes to internal processes, resources, and priorities, while the latter is mainly about accepting different preferences (Bouckaert et al. 2010). This dynamic can lead to a “burden-capacity gap”, as new policies are more easily adapted at the formulation stage at higher levels, while additional resources for coordination and implementation are more difficult to mobilize at lower levels (Fernández-I-Marín et al. 2023).

To overcome these challenges, Peters (2018) suggests establishing networks, particularly among career civil servants; enhancing collaboration in reframing the problem; and utilizing hierarchical authority from central government. Centralizing decision-making at higher levels often seems intuitive for policy makers as an immediate solution, but can lead to higher information costs and resource inefficiencies (Bogumil and Jann 2005). In contrast, more in-depth solutions, such as reframing, are usually not achievable in the short term and depend heavily on the actors involved. The best approach depends on specific contextual factors and stakeholders, necessitating case-by-case evaluation (Peters 2018).

### Administrative traditions and structures in Germany

Historically, the German administration has enjoyed a high status and has partly been seen as a stabilizing force during political upheavals (Sager et al. 2012). It is characterized by hierarchical, sectoral structures, and a strong legal influence, leading to a legalistic approach to policy implementation, especially in the environmental sector (Moss 2004). Since 1970s, extensive environmental legislation, influenced by European standards, has resulted in complex laws and multiple authorities. This system has traditionally emphasized emission-related, conditionally structured regulatory law based on pollution thresholds. It has imposed clear instructions on the administration, often sidelining more flexible and participatory planning tools such as landscape planning (SRU 2007). Scharpf (1970) criticized this rigidity, noting that the bureaucratic structure hinders responsiveness to social and political changes. Galler (2015) also argues that the environmental administration fails to coordinate adequately. She argues that, as a result, environmental information is not merged into a multifunctional view, sectoral objectives are primarily pursued, and instruments are primarily chosen to achieve the objectives within one’s own area of responsibility.

In contrast, cooperative and participatory policies such as the WFD have been rare in Germany, leading to limited experience with participatory processes (both internally and with the public) (Moss 2004). According to Jedicke et al. (2024), environmental administration in particular is marked by a high degree of autonomy of the individual institutions, public and political pressure, and cost concerns, which often foster an inward focus among enforcement actors. This results in the prioritization of measurable and politically prioritized subtasks over cooperative goals, leading to "organized irresponsibility," formalization, and a safeguarding mentality (Bogumil and Jann 2005). The authors also criticize the lack of open-ended opportunities to test innovations, cross-sectoral cooperation, and personal initiative. These could promote administrative innovations but are often rejected due to a lack of legal protection.

Overall, the German civil service’s legalistic and merit-based system supports entrenched professional standards and procedural fairness. However, it also faces challenges such as rigidity, coordination issues, and a suppression of innovative ideas (Reichard and Schröter 2021). Thus, even decades after their introduction, modern European directives such as the WFD continue to pose significant challenges for the German administration.

### The joint implementation of the WFD and HD in Germany

The European Water Framework Directive (WFD, 2000/60/EC) and the Habitats Directive (HD, 92/43/EEC) share the goals of promoting functional ecosystems and enhancing species and habitat diversity. The WFD is considered to be process-oriented, focusing on entire river basins and a good ecological and chemical status of these water bodies by 2027 (Janauer et al. 2015). In contrast, the HD takes a more static conservation approach, targeting specific natural habitat types and species of Community interest, particularly in the Natura 2000 network. For these, a favorable conservation status must be achieved with a ban on deterioration (Rehklau et al. 2022). In Germany, 51% of active floodplains are Natura 2000 sites, leading to spatial and technical overlaps as the HD also addresses abiotic requirements of protected species (Bundesamt für Naturschutz 2014).

In a specific planning project, conflicts arise when WFD dynamization measures negatively impact the conservation status of HD-protected species, such as amphibians affected by altered water regimes through oxbow reconnection. Lower water management authorities (e.g., districts and independent cities) are typically responsible for WFD measures and coordinate with other entities (Umweltbundesamt 2019). Higher water management authorities (e.g., State Ministry for the Environment) further provide broader conceptual planning through River Basin Management Plans, which specify a framework of objectives and measures for the local planning. The same applies from the nature conservation side in the form of HD Management Plans for all Natura 2000 sites.

The actual planning phase involves coordinating hydraulic engineering measures and assessing environmental impacts, ideally with early engagement of approval authorities and the public. Effective coordination between water management and nature conservation departments is essential, occurring through formal and informal channels such as comments, meetings, and inspections (Janauer et al. 2015). However, this coordination often lacks institutional support and uniform guidelines. The German Federal Agency for Nature Conservation merely recommends “timely coordination of sectoral plans” to address conflicting goals and develop joint objectives (Bundesamt für Naturschutz 2014).

Conflicts with HD-protected areas necessitate coordination but also create a legal conflict that can ultimately only be resolved on a case-by-case basis (for a detailed legal comparison see Möckel 2007; Schmedtje et al. 2011; Janauer et al. 2015). If a project is likely to have significant impact on a Natura 2000 site, an environmental impact assessment is required, although this can be waived if a preliminary screening rules out significant adverse effects or if the measures are deemed maintenance-related (Janauer et al. 2015). In the event of a conflict, project organizers can, for example, try to adapt project plans, seek exemptions for climate and flood protection, comply with permit conditions or adjust the HD management plan. Overall, Janauer et al. (2015) conclude that “both the HD and the WFD contain appropriate legal instruments and sufficient discretionary leeway to find environmentally sound decisions in the individual case.”

**Fig. 1:**
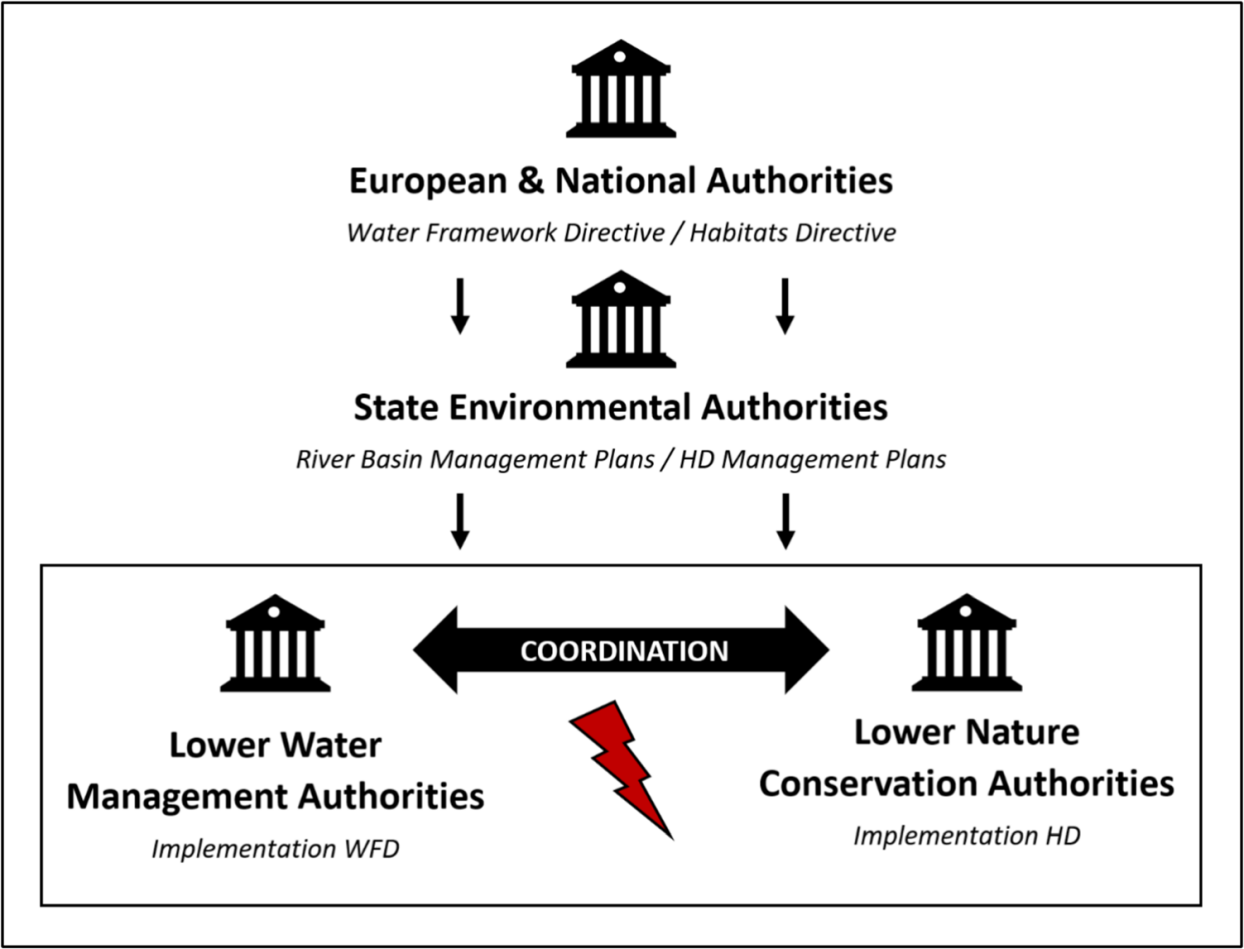
Simplified scheme of administrative authorities in the context of the joint implementation of the Water Framework Directive (WFD) and the Habitats Directive (HD) and the analyzed coordination conflict in Germany

In practice, however, civil servants still face an area of tension, especially from a procedural standpoint. The Umweltbundesamt (2019) also notes that even the authorities involved often lack clarity about early coordination between water management and nature conservation (see Fig. 1). While most cases can be legally categorized and potentially escalated to the European Court of Justice, this is often unattractive for lower administrative levels due to resource demands. Thus, challenges in the joint implementation of the two directives in Germany are likely to stem not only from ecological and legal factors but also from administrative dynamics. The following analysis will therefore examine these challenges posed by the administrative dynamics in more detail.

## Materials and methods

**Fig. 2:**
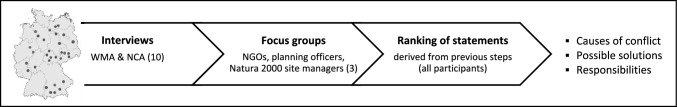
Methodological steps of the study (note: WMA - water management authorities; NCA - nature conservation authorities)

The data were collected in three survey steps including qualitative interviews, focus groups and a quantitative ranking of statements (see Fig. 2). First, we conducted ten interviews with administrative representatives from water management authorities and nature conservation authorities (see Appendices S1 and S2). Experts were selected on the basis of pre-defined criteria corresponding to a targeted case selection, in order to represent diverse administrative competences, federal states, and restoration projects. Participants included individuals from all sub-national levels (state, regional, and local), with a focus on lower authorities, as local levels make independent decisions on WFD measures (Schröder et al. 2020). A representative from a federal authority was also consulted based on prior sub-national experience. Selected experts represented special authorities and regular authorities, and the whole regulatory approval process (e.g., planning, approval, implementation, consultation, and control). At the beginning of each interview, participants estimated potential conflicts between the two directives for their projects, followed by discussions on practical and legal challenges, applied solutions, and recommendations.

Next, we conducted three focus groups with 16 non-administrative stakeholders, including representatives from conservation organizations, planning officers, and Natura 2000 site managers, to gain an external perspective (see Appendices S1 and S3). Participants were selected using the same criteria as for the interviews. Based on the first round of interviews, we developed nine statements about conflict causes, proposed solutions, and institutional responsibilities (see Appendix S4). During the focus groups, the participants ranked these nine statements and discussed the three guiding questions. Both interviews and focus groups were conducted digitally between January and June 2023 with a duration of 60 to 90 min. In total, we covered experiences from over thirty restoration projects across 12 federal states and a variety of restoration measures (see Appendix S1).

In the final consultation round, the nine statements were presented to the previous interview participants for ranking, similar to the focus groups. For the subsequent analysis, we combined the rankings and excluded statements with less than 50% agreement from all participants, thus, narrowing down the main lines of the conflict. An inductive content analysis (Mayring 2010) according to the research questions was applied to the transcripts, with intercoding agreements established to ensure consistency between the two coding persons.

The coded sections were distributed across the identified barriers and categorized into the inductively developed categories of ´institutional´, ´processual´, and ´individual´ barriers. We define ´institutional´ barriers as institutional structures in the form of the official distribution of competences, legal requirements, and standards, which, as overarching ´rules of the game´, define the framework conditions for the implementation process and the individual civil servants. ´Processual´ factors refer to the existence of concrete process structures, procedures, and specifications in the planning and implementation of restoration projects, which influence the interaction between individuals or authorities and the processes of this exchange. ´Individual´ factors include aspects of the individual behavior of actors based on their personal motives, characte,r or education, as well as the influence of social groups on individual decision-making behavior. This categorization outlines the hurdles discussed in the following chapter. Statements by interviewees are indicated with an ‘I’, statements by focus group participants with an ‘F’ (see Appendix S1).

## The conflict of objectives in practice

As explained above, the two directives come into conflict when habitats or species protected under the HD are negatively affected by dynamization measures under the WFD. All interviewees and focus group participants confirmed this fundamental potential for conflict in practice (I1-10;F1-16). However, they also highlighted ways to mitigate the conflict, such as prioritizing planning objectives based on the conservation status and the severity of the impact; temporary loss or exchange of species based on a development forecast; adaptation of construction measures; and active creation of alternative habitats (I1-10;F3,5,9,12–14,16).

However, six persons, also reported on projects with insurmountable conflicts due to physiological conditions (e.g., complementarity of protected species and poor conservation status) or internal administrative obstacles (I2-4,10;F5,13). Several civil servants, deviating from the legal interpretation outlined above, described applied solutions the legal tenability of which was itself questioned under a strict judicial review. “*If everything is legally scrutinized down to the smallest detail, that’s a killer argument*” (I10). These solutions are in the gray area between potential illegality and potential room for interpretation, particularly concerning the assessment of the severity of the intervention, the use of forecasts, and the classification of measures as maintenance (I2-4,8,10). This raises the question of why some individual civil servants are being pushed into these gray areas and what hurdles are complicating the joint implementation of the directives in practice.

The following sections will analyze these obstacles at institutional, processual, and individual levels. It is important to note that restoration projects recorded here are of course also restricted by factors such as land availability, public approval, and limited administrative resources (I1-10;F1-16). While these factors are significant framework conditions, the analysis will focus primarily on additional challenges, as the prior are not the core issue in the specific conflict case discussed.

### Distribution of competences and legal basis at the institutional level

A key issue in joint implementation is the division of competences among the individual authorities (I3,5,9,10;F1,3,4). Most notably, water management authorities’ jurisdiction ends at the mean water line, with floodplains above this level falling under the HD and lower nature conservation authorities. This requires additional coordination for the water management authorities to address a lateral connection of these riparian areas (I3,8). A conservation representative pointed out that this legal division means that cross-sectoral work quickly comes up against legal limits: "*If I agree to a measure that is not based on the HD but on the WFD, I’m technically committing an offence by spending money on something outside my responsibility*" (I9). Several participants suggested creating a legal framework that would allow authorities to invest in objectives across different directives (I3,6,9,10).

The legal basis itself was also partly criticized for being inflexible, with many participants advocating for a coherent adaptation of the two directives at the European level (I2-4,6,8,10;F2,6,7,8,12,14,16). Several persons expressed confusion over why artificially created natural habitat types, such as reedbeds in straightened watercourses, require similar compensation as natural ones, or why motorway constructions have the same legal compensation requirements as restoration projects (I1,3,4,6;F9,13). However, the comments primarily called for selective adjustments to the HD (e.g., including ‘dynamic rivers’ as a habitat type; F8) rather than questioning the directives’ overall ambitions. Almost half of the respondents did not consider the HD to be overly strict per se. Nature conservation representatives in particular stressed the need to maintain the directives´ integrity, especially as an adaptation process of the directive harbors the risk of a fundamental softening of the directive by non-environmental actors (I2;F7,12–14). In addition, certain habitat types, such as HT6510 ‘lowland hay meadow,’ require stricter implementation in Germany due to European infringement procedures (I1,2,10;F8). Thus, both the institutional division of competences and, in part, the logic of the HD itself increase the coordination efforts and the subjective legal uncertainty.

### Lacking coordination and guidance at the processual level

Secondly, a lack of coordination among the authorities hampers an integrative approach (I1-4,6,8,9;F1,3–5,10–12,14). While there is potential for early collaboration in the planning process, this is often not utilized (1,3,4,8;F4,5,14). Water management authorities often do not involve nature conservation partners until later stages of the planning process or only during the permitting phase. This limits their influence on project planning and delays the identification of conflicts, the building of trust and the exchange of specific knowledge (I1,4,9;F5). If changes are only requested retrospectively, additional resources are needed to change previous decisions (I1,3,4,8;F5,14). Conversely, it was criticized that lower nature conservation authorities sometimes refuse early involvement due to a lack of interest in river restoration (I2-4;F5).

Several participants (I4;F2,4,5,14) called for clearer guidelines from higher authorities on communication and collaboration between lower authorities. Although targets are often set by law from above, there are no regulations governing coordination at lower levels (I2,6;F4,5,9,12). In consequence, departments often do not feel responsible for dynamization projects without a clear mandate from above (I2,4;F4,5,9,12,13). Several persons emphasized the so-called Landshut model (cf. Janauer et al. 2015), a pilot project in parts of Bavaria, as a useful template for joint planning processes and guiding the active involvement of stakeholders at local and higher levels (I1,8;F6,9,14).

Additionally, the corresponding specialized planning documents, such as HD Management Plans and River Basin Management Plans, are often poorly coordinated in terms of content and timing or are absent altogether (I4,6,7,9;F2,6,9,12,15). This results in a lack of reference bases for planning assessments or conflicting objectives set by different higher authorities. As mentioned before, if measures for dynamization or the idea of restoration are already outlined in the HD Management Plan, a preliminary project screening may be unnecessary and the scope for dynamization measures is increased (I4,6,8;F2,12). Overall, processual conflicts arise from vague coordination structures and a lack of guidance from higher authorities, which could mitigate issues at the project level.

### Risk avoidance, beliefs, and inflexibility at the individual level

In consequence, most participants emphasized the crucial role of individual civil servants in the success of the process (I1-4,8,9;F2,3,5,8,9,11–13,16). They criticized the limited use of interpretative flexibility in planning and approval procedures (I3,4,9;F1,3,6,8–13,16) and noted that many colleagues hesitate to step outside their familiar, legally secure sphere of influence and knowledge in order to avoid potential legal and career risks (F3,11,13). According to several persons, this applies in particular to younger colleagues, which makes generational change an additional challenge (I3,4;F13). In contrast, more experienced colleagues often find ways to circumvent a supposedly unsolvable conflict (I3,4,8;F12,13). In addition, partly incomplete information about restoration impacts creates unintended interpretative gaps and hinders compromise efforts (I2,6,8;F5,6,12).

Participants noted that the own education, social background, and networks influence how conflicting objectives are perceived (I3,4,9;F2,3,7,8,9,11,14). Some highlighted that, while water management has traditionally focused on construction, the WFD now necessitates greater attention to ecological concerns (I2,3,9;F1,8,14,16). Conversely, nature conservation has tended to focus on static approaches, with process-oriented concepts like wilderness only recently gaining traction again (I2-4,6,9;F5,9,11,13,14). Furthermore, social networks and peer groups would often put pressure on nature conservation authorities to interpret the HD strictly (I9;F1,9,14). Close connections to local nature conservation actors, like local ecological field stations and dedicated voluntary conservationists, to whom a static approach to conservation was repeatedly attributed, would reinforce this tendency (I9;FG9,14).

**Fig. 3:**
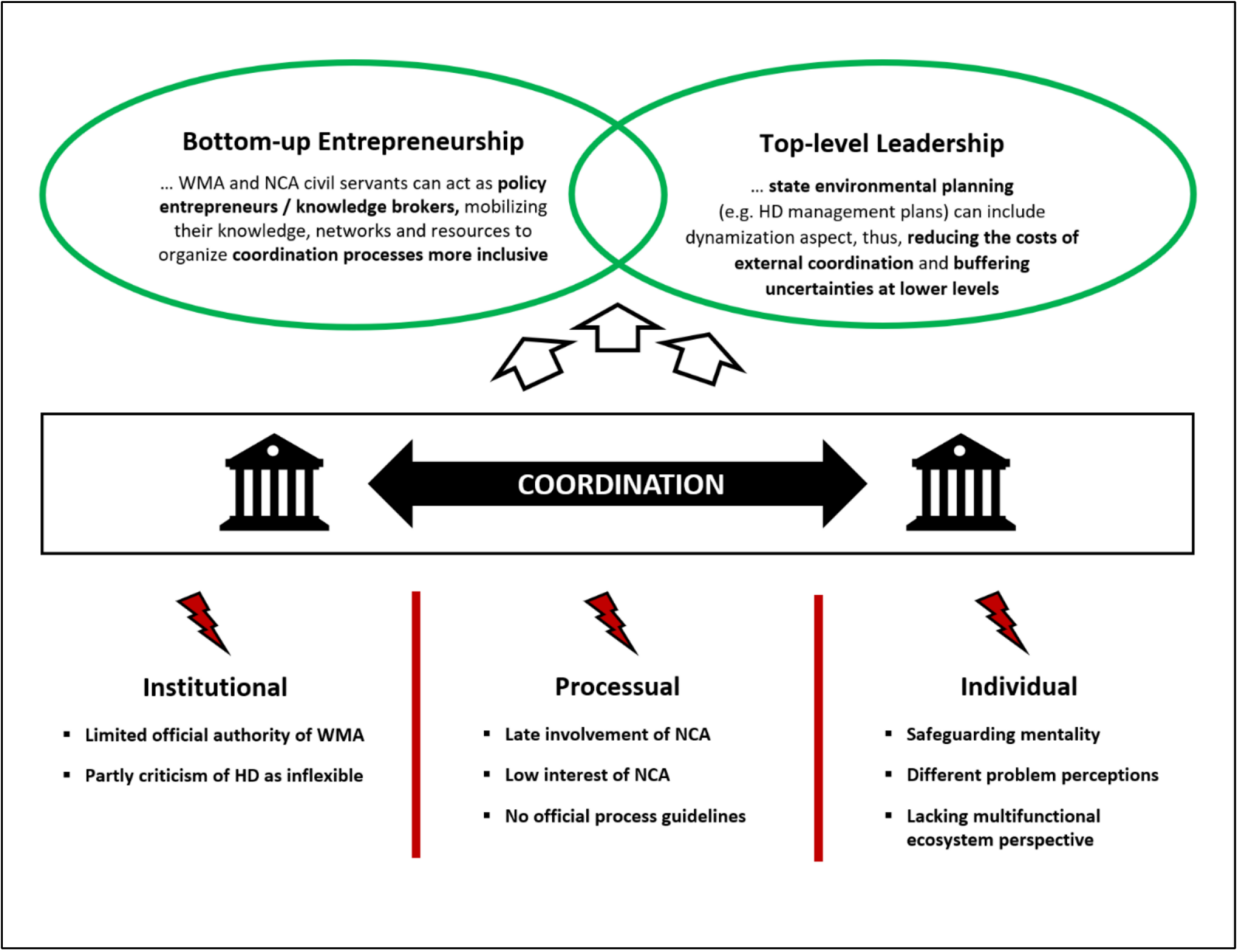
Identified barriers and levers to coordination (note: barriers are indicated by red lightning bolts; levers are indicated by green circles)

Nevertheless, most participants struggled to delineate clear boundaries between the sectors. On the one hand, they called for a more holistic view of ecosystems from both nature conservation and water management colleagues (I2-4,6,8;F1,2,7,9,11,12,14). On the other hand, they also attributed civil servants’ inflexibility simply to their legal training, which would promote a strict application of the law rather than an increased understanding of the ecological value of river restoration. (I3,4,8). Overall, while acknowledging existing sectoral distinctions, participants emphasized the crucial role of individual civil servants in processual outcomes and stressed the need for greater awareness of process-oriented conservation to enhance coordination. Figure 3 summarizes the main hurdles identified and possible levers discussed below.

## Discussion

In the analyzed case, the main incoherencies appear in the implementation process itself, rather than in the directives’ objectives or logic (cf. Nilsson et al. 2012). Despite slightly differing objectives, practitioners confirmed that, in general, joint projects can be realized under these conditions. Thus, supposedly incoherent policies can also be implemented coherently at lower levels, if certain institutional arrangements are in place. From a policy integration perspective, two main solutions emerge in consideration of the public administration literature:

### Bottom-up entrepreneurship offers decentralized support for joint implementation

Firstly, both water management and nature conservation civil servants can act as policy entrepreneurs (Mintrom and Norman 2009) using their knowledge, social networks, and resources to organize coordination processes more inclusively. As the case study shows, successful integration depends on the willingness of individual officials at different levels to push for integration rather than simply adhere to legal requirements. This entrepreneurship depends on a more active role perception of the individual civil servant, ideally equipped with an open mind, good communication and networking skills, and experience in cross-sectoral processes. A key goal is to build trust, or at least the willingness to talk, and to be able to seize the right moment in the planning process for this exchange. It is, therefore, less a matter of making concrete decisions than of creating an openness for a joint search for solutions. Existing social networks, especially with other actors in the other organizational unit or at higher levels, are important to ensure support in the other organizational unit and thus facilitate the willingness of the counterpart to cooperate.

However, as outlined before, in Germany, civil servants are barely incentivized to deviate from these established routines, preferring incremental change over innovation. Sub-national authorities have strict mandates tied to specific directives and, thus, to the intended use of public funds, with little room to consider other directives’ needs (Peters 2015). While this clear allocation of responsibilities prevents downstream inefficiencies, it can also reinforce sectoral silos and raise coordination costs (Peters 2018). This mirrors a collective action problem (Ostrom 1990), where individual actors lack institutional incentives to collaborate, even though the overall administrative process would benefit from alternative behavior. Looser hierarchies and cross-ministerial evaluation could support the tendency in bureaucracy to function less as a strictly directed, executive system, but to find solutions through cooperation and bargaining (Nunan et al. 2012). In addition, joint training or field visits could foster the willingness of different units to collaborate.

In the light of the cases examined, it appears that these policy entrepreneurs are not completely absent from the German administrative landscape. At the same time, given limited resources, understaffed offices and sector-specific training, the scope for individuals to challenge rigid structures in daily administrative routines appears to be limited. Overall, there were no clear indications of the likelihood of such policy entrepreneurs in the projects studied, depending on project size, type, or region. Rather, the overall picture shows a high degree of dependence on individual actor constellations in combination with the hurdles identified. In practice, therefore, lower-level agencies inevitably need more resources and a political mandate to be able to coordinate effectively and make decisions independently. Stronger vertical coordination with higher-level institutions (and granting lower-level officials a certain degree of freedom) can also open up scope for solutions.

### Top-level leadership can provide the political mandate to buffer uncertainties at the local level

Secondly, perceived and institutionalized accountability to higher administrative levels play a crucial role in most administrative systems. Centralized decision-makers, ministries, or specialized planning bodies can reduce coordination costs by making overarching decisions for local implementation (Kaplaner et al. 2023). For the WFD and HD, incorporating aspects of dynamization into state-level plans (e.g., HD Management Plans), can—complementary to the requirements of the directives—provide a mandate for stronger coordination, clarify responsibilities, and maintain case-specific flexibility. More participatory models, such as the Landshut model in parts of Bavaria, may be interpreted as initial responses to this approach. However, shifting the decision-making requires effective information exchange from the bottom (implementation stage) to the top (formulation stage) (Fernández-I-Marín et al. 2023). The challenge lies in the necessary compression of information along vertical communication channels, which requires comprehensive cross-level exchange (Bogumil and Jann 2005). In principle, both paths can lead to success, but combining bottom-up initiatives with top-level support offers the greatest chance (Cejudo and Trein 2023a).

This case study strongly reflects the aforementioned tradition of sectoral and hierarchical structures in German administration. Interviewees suggested that the nature conservation administration is inclined in particular toward legalistic policy implementation. According to Jedicke et al. (2024), this strict implementation mentality is driven by the legal framework, which necessitates strict enforcement of conservation goals, and a fundamentally welcome intrinsic motivation to preserve individual species, which can, however, lead to a confrontational attitude. Thus, the case demonstrates the need for greater awareness of holistic ecosystem considerations among officials in the long term, in order to achieve an integrative framing in all sectors concerned.

This need may apply not only to the sub-national level, but also to the level of European legislation. Although the directives themselves have not been identified as the core cause from a sub-national perspective, a better coordination of their logic of protection and implementation also seems to make sense from a European level perspective. This need for reflection may concern both the legal structure and (analogous to sub-national units) the European administrative units responsible for the directives and their actors themselves. At the same time, it seems to make sense to take greater account of the necessary scope for local implementation at the European level when drafting or revising environmental directives. The WFD in particular has provided an impetus for this in recent decades, supporting a trend toward more cooperative approaches and more holistic ecosystem perspectives. This also reflects a certain basic sectoral tendency, despite the aforementioned great importance of individual officials.

It should be noted, however, that despite the problems described, positive examples of cooperation and counteracting path-dependency have emerged, with proactive individuals on both sides seeking cross-sectoral solutions. The mindset, especially around the WFD, seems to be moving faster in this direction, although limited resources, guidance, and mandates are slowing progress. The new European Nature Restoration Law offers further opportunities for all departments concerned to drive this modernization process forward.

From a theoretical perspective, the results emphasize the growing role of individual actors and integrative leadership, highlighting the processual nature of policy integration (Cejudo and Trein 2023b; Lambelet 2023). The case study illustrates the asynchronous and stage-dependent nature of policy integration (Candel and Biesbroek 2016), thus, underscoring the need to distinguish more precisely between integration processes in the political and administrative spheres, as well as integration processes between them. Coherent integration and implementation in administrative systems may have different dynamics than in broad day-to-day political processes. For instance, accountability in administrative contexts, while sometimes restrictive, can be more beneficial in political contexts (e.g., holding countries accountable to international biodiversity targets).

With regard to these findings, it must be considered that this study provides insights based on actors’ perspectives. Thus, it does not prove that addressing the identified barriers would achieve integrated policy processes. However, including different stakeholder groups allowed for a nuanced understanding of the main lines of the conflict. Furthermore, the survey focused solely on the German sub-national environmental context, examining intrasystemic-intrapolicy integration, i.e., the observation of two thematically related (environmental) policies within one political system (Bornemann 2014). A similar study in other member states or across different environmental directives could identify additional factors and clarify whether these challenges are specific to German federalism.

## Conclusions

The joint implementation of the WFD and HD in Germany confirms that policy integration and coordination are crucial to overcome fragmented administrative structures, as noted by Lidén and Nyhlén (2023). Shifting the focus from ecological and legal aspects to institutional and administrative factors revealed additional barriers and areas for improvement within administrative structures and at the individual level. We found that the failure of river restoration projects in Germany stems not only from practical hurdles (e.g., resources, information, or manpower), but also from more fundamental flaws in the administrative arrangements. Existing policy incoherencies are primarily found in implementation structures, rooted in institutional, processual, and above all individual factors.

This perspective offers a more holistic understanding of the conflict and can aid further implementation of the directives. Key recommendations for administrative practice include:

- **Bottom-up entrepreneurship**: Civil servants in water management and nature conservation can act as policy entrepreneurs, using their knowledge, networks, and resources to facilitate inclusive coordination. This requires sufficient administrative resources, flexible implementation mandates, and reframing of the issue.
- **Top-down leadership**: State-level planning, such as HD Management Plans that incorporate dynamic river development, can provide mandates and legal clarity, reducing coordination costs and ensuring greater legal security at the local level.
- **Multifunctional conservation perspective**: Similar to the exchange with local land managers and stakeholders, common ground and goals have to be developed within the environmental authorities as well. Over time, river conservation must be reframed to recognize river ecosystems as multifunctional and dynamic, which does not require major structural reforms but a shift in understanding. Joint training, project exchanges, and external training opportunities can foster awareness of integrative approaches.

While transferring the decision-making to higher levels appears to be a short-term solution, in the long run, it is especially important to focus on reframing the overall problem and establishing robust coordination mechanisms.

Germany’s tradition of sectoral and hierarchical environmental administration makes flexible implementation challenging, revealing a meta-conflict between process-oriented and static conservation approaches in this specific case. River restoration by its very nature requires a loss of control and unpredictability, which conflicts with the traditional administrative mindset of security and predictability. While modern European directives continue to challenge this tradition, there has also been some progress in adopting a more flexible planning attitude, particularly in the context of the WFD.

From the theoretical perspective of policy integration, the case highlights:

- The critical role of **individual actors and leadership** at various political and administrative levels for successful policy integration.
- The **dual nature of accountability** in administrative systems, which can prevent downstream inefficiencies, but also reinforce sectoral silos and increase coordination costs.
- The value of **cross-sectoral coordination and vertical integration** (e.g., higher-level planning) to support joint implementation.

At the moment, the ultimate question is whether existing coordination mechanisms and actors can overcome policy incoherencies by practically weighing up options for action, even in absence of overarching prioritization. While this may lead to successful, legally compliant processes in some cases, higher political levels must assess whether the current reliance on individual civil servants and the additional coordination costs provide a satisfactory framework for future restoration governance.

As many other EU member states, Germany is unlikely to meet the WFD´s requirements by its termination in 2027 (Moss et al. 2020). As outlined above, this failure is not merely due to resource constraints but also stems from policy incoherence and fragmented administrative structures. To improve the joint implementation of environmental policies, EU and national policymakers must focus more carefully on the sub-national level, providing the necessary mandates and resources to address these issues.

## Supplementary Information

Below is the link to the electronic supplementary material.

Supplementary file1 (PDF 1021 kb)

## Data Availability

The data that support the findings of this study are available from Helmholtz-Centre for Environmental Research (UFZ), but restrictions apply due to privacy issues and so data are not publicly available. The data are, however, available from the authors upon reasonable request.
