# Supplementary material for: Living with the incoherent: Practical insights on implementing European restoration policies for biodiversity policy integration
Source: Ambio. 2025 Apr 19;54(10):1635–47. doi: 10.1007/s13280-025-02180-2 (PMC12405081; doi:10.1007/s13280-025-02180-2)

Ambio

Supplementary Information

This supplementary information has not been peer reviewed.

Title: **Living with the incoherent: Practical insights on implementing European restoration policies for biodiversity policy integration.**

Appendix S1 - Overview of interview partners and participants of the three focus groups (NCA = nature conservation authority; WMA = water management authority)

Interviews					
**Nr.**	**Type of authority**		**State**	**Project types**	**River Systems**
I1	Middle authority	NCA	Bavaria	River widening; dyke relocation; flood protection measures	Danube, Lech, Iller
I2	Higher authority	NCA	North Rhine-Westphalia	Slotting of embankments; damming of drainage ditches; creation and reconnection of floodplains	Lippe, Ruhr
I3	Lower authority	WMA	Saxony-Anhalt	Dyke relocation; reconnection of oxbows; habitat maintenance measures	Havel, Elbe
I4	State technical authority	WMA	Brandenburg	Reconnection of oxbows; connection of flood channel; habitat maintenance measures; installation of groynes	Spree
I5	State technical authority	NCA	Saxony	Reconnection of floodplains; revitalisation of riparian forests; dyke relocation; slotting of embankments; flood protection	White Elster, Luppe, Zwickauer Mulde, Elbe
I6	Lower authority	WMA	Mecklenburg Western Pomerania	Dismantling of weirs; reconnection of floodplains; reconnection of oxbows	Warnow, Nebel
I7	State technical authority	WMA	Rhineland Palatinate	Dyke relocation	Nahe, Holzbach
I8	Lower authority	NCA	Bavaria	Reconnection of oxbows	Danube
I9	Federal technical authority	WMA	Hannover	Projects of the Federal Government's Blue Belt Programme (*Blaues Band Deutschland*)	Weser, Aller

I10	Lower authority	NCA	Saxony-Anhalt	River widening; dyke relocation	Elbe

Focus Groups

Nr.	Type of organisation	State	Project types (selection)	River Systems (selection)
F1	Site Management	Bremen	Restoration of rivers and floodplains	Wümme
F2	Site Management	Bavaria	Restoration of rivers and floodplains	Isar
F3	Site Management	Hamburg, Lower Saxony	Restoration of rivers	Elbe
F4	Site Management	Baden-Wuerttemberg	Dyke relocation	Elbe
F5	Site Management	Thuringia	Restoration of rivers and floodplains	Hasel, Lauter, Werra
F6	Planning Offices	Bavaria	Restoration of floodplains	Danube, Inn, Isar
F7	Planning Offices	Thuringia	Planning and implementation of restoration projects; construction supervision; watercourse development	Gera, Wipper
F8	Planning Offices	Lower Saxony	Dyke relocation	Elbe
F9	Planning Offices	North Rhine-Westphalia	Restoration of rivers and floodplains; planning and implementation of restoration projects; construction supervision	Not specified
F10	Planning Offices	Saxony	Reconnection of oxbows; watercourse development; restoration of riparian forests	Mulde
F11	Planning Offices	North Rhine-Westphalia	Restoration of rivers and floodplains; ecological assessment of watercourses	Danube, Main, Salzach, Elbe, Isar

F12	Non-governmental organisation (local)	Brandenburg	Dyke relocation; revitalisation of riparian forests	Elbe, Hohe Garbe
F13	Non-governmental organisation (regional)	Saxony	Restoration of rivers and floodplains; communication to the public; establishment of integrative land use	Luppe
F14	Non-governmental organisation (national)	Bavaria	Removal of transverse structures and dams; planning support for restoration projects	Ammer, Danube, Isar
F15	Non-governmental organisation (national)	Berlin	Planning support for restoration projects; communication to the public	Not specified
F16	Non-governmental organisation (regional)	Thuringia	Restoration of rivers and floodplains; revitalisation of riparian forests	Saale

Appendix S2 – Interview guide

Interview guide

Introduction & project description

1. Please briefly outline your professional background in the context of water management and in which projects you are currently involved in this field.
    - What are the primary objectives of these projects?
2. What do you see as the fundamental challenges for the dynamization of rivers and floodplains?

Block I - Causes of conflict (focus: Natura 2000 sites)

3. Which conflicts of interest, in particular between static-oriented and process-oriented positions, occur in your projects?
4. What are typical (legal/technical) obstacles to restoration projects focusing on dynamic processes?
5. Which natural habitat types and species of Community interest or other species relevant to nature conservation specifically occurring in watercourses and floodplains are affected in your projects?

Block II - Applied solutions (focus: Natura 2000 sites)

6. What approaches have been/are being used in your projects to achieve practical solutions that are suitable for authorization under nature conservation law?
7. Who do you see as the key players in this area of tension?
    - Who is driving dynamization? How / why?
    - Which actors are resisting? How / Why?

8. How did the dialogue between these stakeholder groups take place to find possible solutions to the conflicts?
    - When did this dialogue take place?

Block III - Possible improvements in the field of conflict

9. How could planning and administrative action be facilitated in future to implement dynamic watercourse and floodplain development in Natura 2000 sites in a legally secure manner?
10. What procedures and instruments could support this?
11. Where do you see positive developments/examples?
12. What political or administrative framework conditions are necessary for this?

Closing question

13. If you had a magic wand that could do anything, what would you do to make the dynamization of watercourses and floodplains in Germany easier possible? Please name one idea that comes to mind spontaneously.

Appendix S3 – Guide for focus groups

Focus groups guide

Duration	Thematic block
**10min**	**Introduction**
	• Personal introduction<br>• Introduction of the background of the study and the agenda of the focus group<br>• Short round of introductions by the participants
**5min**	**Ranking of nine statements**
	• Short introduction of the nine statements (conflicts, solutions, responsibility)<br>• Online ranking of the statements by the participants<br>• Short display of results
**~ 20min**	**Block I - Causes of conflict (focus: Natura 2000 sites)**
	• Short reflection of the results of the ranking (conflicts)<br>• Open discussion of project experiences<br>• What are the main lines of conflict?
**~ 20min**	**Block II - Possible improvements in the field of conflict**
	• Short reflection of the results of the ranking (solutions)<br>• Open discussion of project experiences<br>• How can these conflicts be solved?
**~ 20min**	**Block III – Responsibilities of actors**
	• Short reflection of the results of the ranking (solutions)<br>• Open discussion of project experiences<br>• Who is responsible for implementing these solutions?
**5min**	**Conclusions**

	• Short summary of discussed main aspects<br><br>• Feedback by the participants and final remarks

Appendix S4 – Visualised ranking of the nine statements by the interview partners and participants of the focus groups regarding the causes of the conflict, possible solutions and responsibilities (joint display; n = 24; scale: dark red (-2) = strongly disagree; light red (-1) = somewhat disagree; grey (0) = indifferent; light green (+1) = somewhat agree; dark green (+2) = strongly agree)

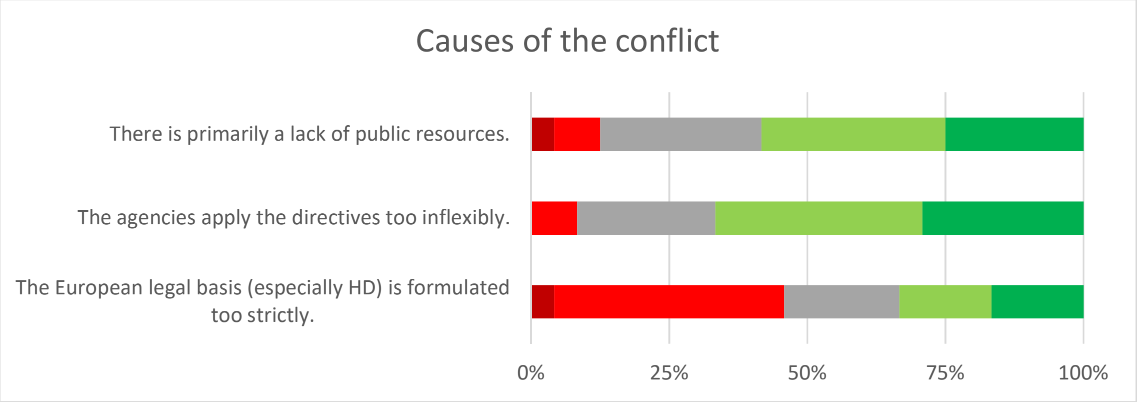

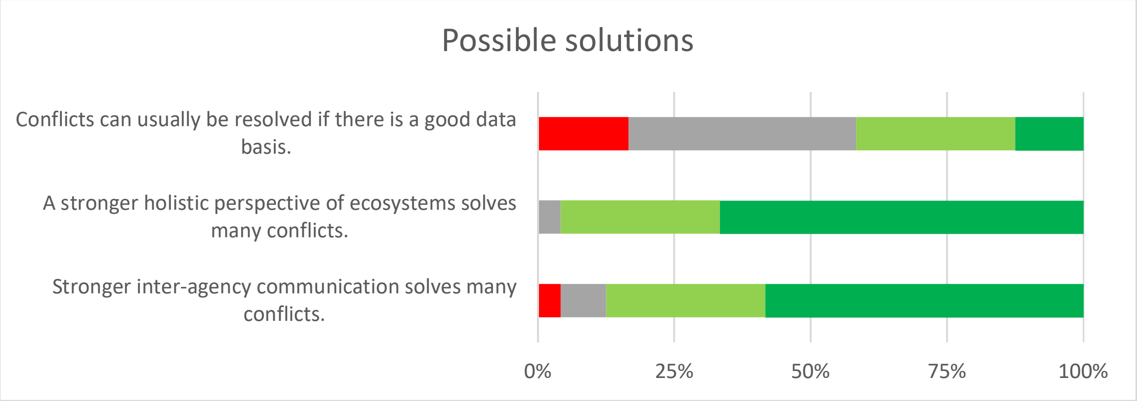

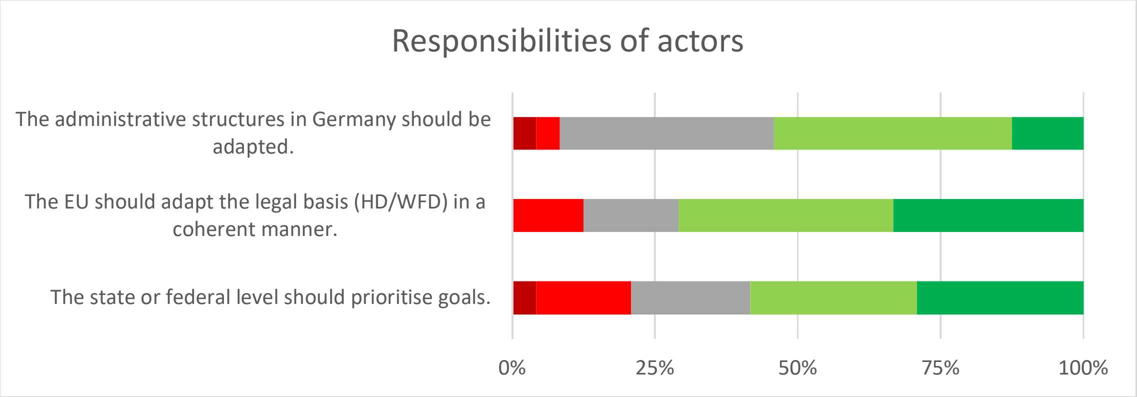

Supplement: Supplementary file 1 — Supplementary file1 (PDF 1021 kb) [file 13280_2025_2180_MOESM1_ESM.pdf]
